# Content validation of the teacher food and nutrition-related health and wellbeing questionnaire, a Delphi study

**DOI:** 10.1186/s12889-025-22555-0

**Published:** 2025-04-21

**Authors:** Tammie Jakstas, Tamara Bucher, Andrew Miller, Vanessa. A. Shrewsbury, Clare. E. Collins

**Affiliations:** 1 School of Health Sciences, College of Health, Medicine and Wellbeing, The University of Newcastle, Callaghan, NSW 2308 Australia; 2 Food and Nutrition Research Program, Hunter Medical Research Institute, New Lambton Heights, NSW 2305 Australia; 3 School of Environmental and Life Sciences, College of Engineering, Science and Environment, The University of Newcastle, Callaghan, NSW 2308 Australia; 4 School of Health Professions, Bern University of Applied Sciences, Bern, Switzerland; 5 School of Education, College of Human and Social Futures, The University of Newcastle, Callaghan, NSW 2308 Australia; 6 Priority Research Centre for Teachers and Teaching, The University of Newcastle, Callaghan, NSW 2308 Australia

**Keywords:** Delphi, Nutrition, Teachers, Wellbeing, Questionnaire, Determinants of nutrition and eating (DONE Framework).

## Abstract

**Background:**

Schoolteachers’ personal health and wellbeing are priority to ensure quality teaching, positive student outcomes and improving teacher retention. With limited-availability of validated tools to measure teacher food and nutrition (FN) as a component of wellbeing, this study aims to evaluate the content validity of the Teacher Food and Nutrition-related health and wellbeing Questionnaire (TFNQ) to fill this gap.

**Method:**

A two-round Delphi was used to refine the questionnaire and establish content validity. Round-one reviewed constructs and lifestyle covariates (LC) for inclusion. Round-two considered construct phrasing, appropriateness of scales and questionnaire order. Descriptive and thematic analyses were conducted.

**Results:**

Round-one included 23 international experts, with 19 also participating in round-two. After round-one, seven constructs and three LC were removed with two constructs revised into four concise new groupings to refine the TFNQ. In round-two 83% of experts agreed with questionnaire order, and feedback indicated only minor adjustments. The final TFNQ contains 26 FN and wellbeing constructs and three LC.

**Conclusion:**

This Delphi study established content validity of the TFNQ to appropriately measure key aspects of FN as a component of wellbeing in schoolteachers. Future testing will evaluate the TFNQ construct validity and reliability.

**Supplementary Information:**

The online version contains supplementary material available at 10.1186/s12889-025-22555-0.

## Background (5590/ 8000)

Primary and secondary schoolteachers are important Influencers in the development of students’ food and nutrition (FN) practices, making their wellbeing a unique area of concern as the influential effects are two-fold affecting both the teacher and their students. Teacher health and wellbeing contributes to their ability to influence student nutrition, health, and wellbeing outcomes as educators and role models, and is identified as an important factor in teacher attrition associated with workplace stress and burnout [1]. Additionally, concern has been expressed regarding limited teacher knowledge of nutrition, weight-related health, and chronic disease [2], known to be factors influencing teacher self-efficacy in the delivery of nutrition education [3]. The influence of teacher health and wellbeing on student outcomes and teacher retention [4], warrant further consideration in understanding how FN practices impact teacher wellbeing and their ability to be positive FN Influencers.

Teaching is a profession where individuals experience elevated levels of stress, anxiety, depression, and professional burnout [5]. In assessing teacher FN, the influence of the school environment and teachers place within it should be key considerations. However, currently, there is a scarcity of validated tools to screen these additional factors [6], with recent reviews on teacher wellbeing interventions acknowledging limited inclusion or screening of teacher FN practices [7]. A teachers’ personal FN (Table 1, Additional file 1) factors are influential in helping fulfil their professional FN (Table 1) roles and responsibilities as gate keepers, educators, health promoters and role models of healthy FN practices [3, 8]. What teachers do inside the classroom as educators and gate keepers [8], and the impact of a teachers’ actions beyond the classroom including dieting practices and food or weight-related behaviours [9], can impact their ability to be effective role models and health promoters [10]. Without first equipping and supporting teachers to achieve good personal FN wellbeing, how can they be expected to support and positively influence the FN wellbeing of students?

**Table 1:** Teacher personal and professional food and nutrition defined

Personal FN	Factors related to an individual teacher, i.e., diet quality, food skills, food attitudes and perceptions.
Personal FN Constructs	A group of questions, or scale that measure an aspect of teachers’ personal FN such as diet quality, food and cooking skills, food attitudes and behaviours
Professional FN	Factors specifically related to a teachers’ professional role, i.e., classroom FN practices, role modelling food behaviours, health promotion and FN educator.
Professional FN Constructs	A group of questions or scale that measure a teachers’ professional FN attitudes, knowledge, or practices such as classroom FN practices, nutrition teaching self-efficacy or capacity to role model healthy FN practices (i.e., healthy eating).

Legend: FN = Food and Nutrition

To improve the health promoting capacity of schools, researchers have identified that contemporary school health programs should include the health of school employees, to potentially aid teacher retention, productivity, positively impact education quality and the health of students [4]. Additionally, the concept of food wellbeing has been explored to consider the idea that food is not just medicine but a way for individuals to connect with community, culture, and families [11] providing an alternate view for exploring teacher wellbeing.

The idea that food consumption can influence wellbeing beyond just nutrition, is an integrated approach that allows consideration and inclusion of the many determinants influencing an individual’s healthy eating practices [12]. Determinants within this paper are defined as the factors of influence on an individual’s food, nutrition and eating practices, with reference to the Determinants of Nutrition and Eating (DONE) framework [13]. Increasingly, research evaluating potential relationships between diet quality, cooking confidence and mental health outcomes are informing holistic interventions to assess the complexity of FN, including integrative culinary practices [14]. This lifestyle medicine approach encompasses the diversity of FN knowledge, skills, attitudes, and behaviours of individuals to select, prepare and understand the connection between foods they eat and the impact on their nutritional status, health, and wellbeing.

Teachers are ideally placed to role model positive lifestyle behaviours. Teacher FN education is beneficial in improving their own health and wellbeing outcomes, such as weight loss [15]. With flow on effects for teacher professional outcomes, including improved classroom FN practices and self-efficacy in the delivery of nutrition education [3]. However, the ability to develop targeted, informed, and effective education interventions for teachers is limited by the lack of information available on the status of teacher FN practices, and studies using validated tools to gather these data.

A recent scoping review mapped the diversity of measures used in research studies globally that explored aspects of FN in teachers and the FN constructs used most frequently [6]. However, at present, no questionnaire exists to comprehensively measure the different dimensions that impact a teachers’ overall FN-related health and wellbeing. Further, none of the existing measures include a comprehensive assessment of FN practices like food skills or cooking confidence which have been shown to be associated with wellbeing outcomes including stress, depression, and anxiety [14, 16].

Therefore, the aim of the current study was to establish content validation with a group of international experts from multiple disciplines, of the Teacher Food and Nutrition-related health and wellbeing Questionnaire (TFNQ), developed to measure diverse FN practices alongside teacher work-related burnout, stress, coping, and personal wellbeing.

## Methods

### Study design

This study was implemented in two stages described below and summarised in Table 2. Stage one involved drafting the initial TFNQ, with stage two being a Delphi study to evaluate content validity.

**Table 2:** Overview of teacher food and Nutrition-related health and wellbeing questionnaire (TFNQ) development and methodology used

Step	Action	Method	Purpose/Action
**Stage One- Drafting of the initial version of the TFNQ**			
1.1	Review FN constructs across global teacher populations	Scoping review	Identify the range of FN constructs previously used in the peer-reviewed literature and potential gaps within teacher FN research.
1.2	Correlates and determinants of healthy eating practices in teachers	Literature search	Identify strong correlates, or FN constructs with an association of interest with dietary outcomes and mental health/wellbeing, and key determinants relevant to teachers in Australia.
1.3	The Determinants of Nutrition and Eating (DONE) Framework	Determinants of FN investigated	Framework used to guide selection of constructs and LC.
1.4	Draft questionnaire	• Collate findings steps 1.1–1.3 • Research team discussions	Development of the initial version of the TFNQ.
**Stage Two- e-Delphi to evaluate the content validity of the TFNQ**			
2.1a	e-Delphi recruitment and participant consent	An online survey using the QuestionPro software	1. Fifty-nine invitations were sent via email with embedded survey link to obtain participant consent to participate. Sent to participants in August 2022.
2.1b	e-Delphi round one	An online survey using the QuestionPro software	1. Assess the value of constructs and LC to obtain valuable information. 2. To establish constructs and LC for removal. Sent to participants in September 2022, with a fortnight provided to complete
2.2	Research team review meeting/discussion	Online team meeting utilising the online meeting platform Zoom (Zoom video Communications Inc, San Jose, CA).	Considered constructs and LC with close consensus vote and/or conflicting comments. Removed and refine constructs and LC for round-two review. All members of the research team (TJ, CEC, VAS, TB, AM) participated in a two-hour online discussion.
2.3	e-Delphi round two	An online survey using the QuestionPro software	1. Refine question phrasing. 2. Assess scale suitability. 3. Assess questionnaire flow (i.e., suggestions for construct/LC order). Sent to participants in October 2022, with a fortnight provided to complete.

Legend: FN = Food and Nutrition, LC = Lifestyle covariates, TFNQ = Teacher Food and Nutrition-related health and wellbeing Questionnaire

## Stage 1

### Review of the food and nutrition constructs used across global teacher populations; and key learnings from the scoping study

A scoping review [6] mapped previous research which demonstrated the diversity of FN constructs used previously and how they had been defined. The review identified questionnaires (94% of papers) as the favoured data collection method. Dietary intake and nutrition knowledge were the most common constructs measured, consistent with the 2002 findings of Contento et al. who examined measures used in nutrition education interventions [17]. Yet, while diet quality as a measure of dietary intake has a strong association with a variety health-related outcomes [18], nutrition knowledge is acknowledged as a weak correlate of health and wellbeing outcomes [19].

Limited studies within the scoping review measured culinary constructs such as teacher food preparation habits or cooking practices (5% of papers). Similarly, mental health, quality of life and wellbeing constructs were sparsely measured across included studies (11% of papers), with limited exploration of the relationship between FN factors and wellbeing outcomes. These findings highlighted research gaps that need to be addressed in teacher populations, such as exploratory studies that investigate the connections between food, nutrition, and culinary practices with diet quality and mental health-related outcomes [14, 20]. Finally, physical activity (41% of papers), followed by smoking status (22% of papers), alcohol intake (11% of papers) and sleep (5% of papers) were the most common lifestyle covariates (LC) measured across included papers [6]. The lack of consistent construct terminology and psychometric testing across the questionnaires included in the review, highlighted the need for the use of clearly defined constructs, and adequate psychometric testing in the TFNQ development.

### Identifying food and nutrition constructs associated with healthy eating practices

The complexity of FN-related health and wellbeing was explored to identify constructs where a clear determinant, correlation or association of interest existed with either diet quality, or measures of wellbeing, as the specific health-related outcomes of interest. This screening process reflected on previous food, nutrition, health, and wellbeing research conducted, including research that reviewed the barriers and facilitators of healthful lifestyle practices of teachers, including healthy eating [21]. Teacher-identified barriers to healthy lifestyle practices within schools, such as workload, leadership, and colleague support were therefore included as constructs for consideration in the initial TFNQ.

### Behaviour change theories and/or health care frameworks considered, to guide construct and lifestyle covariate selection

A wide variety of behaviour change theories and health implementation frameworks were reviewed, with the DONE framework selected to guide the development and final selection of constructs and LC for inclusion within the initial TFNQ [13]. The DONE framework has 51 determinant groups incorporated across four broad categories of influence from individual, interpersonal, environmental and policy that influence eating behaviours and food choices [12, 13]. The interactive nature of the framework enables within category review of specific determinants with reflection of their population level of effect and potential modifiability [12, 13]. The DONE framework therefore enabled the selection of constructs that would be useful to measure and inform education programs or interventions with the potential to affect positive change, at a group or population level to improve eating behaviours for better health and wellbeing outcomes.

### Drafting the teacher food and nutrition-related health and wellbeing questionnaire

Following steps 1.1–1.3 the initial version of the TNFQ was developed with inclusion of other relevant scales, including diet quality using the Fruit And Vegetable Variety (FAVVA) index [22], Eating Social Norms at School scale [23], and the SF-12 to measure individual quality of life [24]. Some constructs applied to the TFNQ were sub-scales drawn from larger measurement tools such as Food Skills Confidence [25], Cooking Attitudes and Self-efficacy from within the cooking and food provisioning action scale [26], and the Resilience and Resistance Eating Practices from the self-perceived food literacy scale [27]. Additional question groups were developed to create new constructs, including the Eating and Food Behaviour at School construct, to fill gaps in areas of interest specific to the school environment in which teachers work. With the General Health Perceptions, and Perceptions of Diet Quality constructs developed to measure determinants identified by the DONE framework with a high level of impact within the population and/or with potential modifiability [12, 13]. Overall, the initial TFNQ included 27 constructs and six LC.

## Stage two

### Expert e-Delphi methodology to refine the initial teacher food and nutrition-related health and wellbeing questionnaire content and establish content validity, round-one

An electronic/online-Delphi (e-Delphi) methodology was used to obtain feedback from a range of experts across different disciples to achieve group consensus and establish content validation of the proposed food, nutrition, health, and wellbeing constructs and LC that would be included in the TFNQ. To establish content validity of the proposed constructs and LC, experts were questioned regarding the degree to which these constructs would provide data of relevance to the intended purpose of the questionnaire. The main purpose of the TFNQ being to assess FN as a component of health and wellbeing in schoolteachers, and the capability of these constructs to measure different aspects of food, nutrition, health, and wellbeing. The e-Delphi provided a methodology that allowed for the systematic gathering, collation, and review of expert feedback, with anonymity of experts to each other’s responses, and the use of structured questions to obtain and evaluate group feedback through the calculation of a consensus vote [28]. In acknowledging that FN practices are complex and multifaceted it was essential to recruit experts from across different disciplines of education, dietetics and nutrition, FN behaviour, and psychology.

Using an online format meant both international and national experts could be invited to participate. Experts from multiple disciplines were identified either through previous collaborations, or as authors of papers included in the scoping review or from literature searches conducted in stage-one [6]. Participants were invited to participate from the disciplines of education, dietetics and nutrition, FN behaviour (including culinary nutrition and food behaviour research), and psychology, with access to the internet, and who had agreed to participate in both e-Delphi consultation rounds. While no a-priori sample size is required within a Delphi methodology [29] a balance of experts was sought across the four discipline areas with the aim of recruiting around five participants for each TFNQ context area. Age, gender, and ethnicity of the participants was not sought as it was their expertise within the specific fields of interest that was important to the success of the Delphi with emphasis on ensuring a balance of expertise from across all included disciplines to provide a well-rounded consensus judgement of the questionnaire content as fit for purpose.

E-Delphi survey questions were structured to obtain a quantitative count or consensus, with expert opinion and experiences in questionnaire development and insight into the different disciplines sought through open-ended style questions. Open-ended questions were reviewed to identify themes of agreement and/or collate feedback on alternative or additional constructs to consider. The 2022, QuestionPro Survey Software (QuestionPro Inc, Austin Tx), was used to distribute and collate both surveys. Data analysis was conducted using Microsoft Excel with results displayed in table format in Microsoft Word.

The e-Delphi methodology used was informed by the original Rand paper [28] and updated research guidelines for Delphi methodology [29], with previous Delphi papers within health science research reviewed [30]. All experts were blinded to the groups’ opinions, with a brief summation of the first-round results discussed in the opening of round-two. Limited feedback was provided to experts, as the aim of round-two did not require extensive feedback from round-one and in consideration of reducing participation burden.

A fortnight was provided to complete the tasks in each round, with both surveys sent via an online link embedded within an email to each participant, and three reminders sent throughout the fortnight, to participants who had not completed a response. Each round was estimated to take approximately 30–40 minutes to complete. With a fortnight between the completion of round-one and start of round-two.

In round-one participants were asked to complete two tasks. First, they were asked to indicate if they agreed, mostly agree, disagree or were unsure, whether the constructs and LC presented in the TFNQ would provide useful information to achieve the purpose (Table 3 provides a description of each option). Population specific screening constructs of Demographic, Teacher Characteristics and Additional FN Education were reviewed individually to select the most suitable screening questions required for a teacher population. Experts were also provided the opportunity to give feedback on alternate constructs and/or LC that could be considered, and where amendments could be made. The second task in round-one involved participants using a rank question to indicate the top three FN, and wellbeing constructs, along with the top three LC to remove.

**Table 3:** Response options and explanations provided to e-Delphi participants

Response	Explanation provided
Agree	The constructs will serve the purpose of the questionnaire
Mostly Agree	Some amendments or item changes are required
Disagree	Questions included will not provide useful data
Unsure	Not my field of expertise

### Research team review meeting

An online Zoom meeting (Zoom video Communications Inc, San Jose, CA) to review round-one results and discuss where expert qualitative feedback conflicted with consensus vote was conducted to finalise the areas of focus for round-two.

### E-Delphi round-two

For each construct, not including those with existing validation such as the Fruit And Vegetable Variety (FAVVA) Index [22], experts were asked to review question phrasing and suitability of scales assigned. The TFNQ item order was also reviewed by either selecting agree or do not agree and providing suggestions for alternate ordering to improve teacher participant experience.

### Data analysis and statistical analysis used

Basic descriptive statistics, including summation of votes to ascertain consensus decisions, with agree and mostly agree summed together, and unsure votes and blank responses removed from the count was used to better obtain a consensus vote for each item.

## Results

### E-Delphi recruitment and participant consent

Fifty-nine experts were approached across the four disciplines with 23 experts from Australia (*n* = 14), Canada (*n* = 1), Switzerland (*n* = 3), United Kingdom (*n* = 2), New Zealand (*n* = 1) and United States of America (*n* = 2) agreeing to participate. The 23 participants in round-one, had expertise across the disciplines of FN behaviour (*n* = 8), education (including tertiary, secondary, primary settings) (*n* = 6), psychology (*n* = 4), dietetics and nutrition (*n* = 5). Eighteen experts in round-one identified previous experience in questionnaire or screening tool development. Round-two included 19 participants from round-one.

### E-Delphi round-one

#### Constructs and lifestyle covariates

I. Consensus vote on the value of constructs and LC

Table 4 provides an overview of the consensus votes achieved in round-one for all 27 constructs and the six LC proposed in the initial TFNQ. In round-one all achieved a consensus above the 75% cut-off set a-priori, indicating agreement on the value and suitability of proposed constructs and LC to screen different aspects of food, nutrition and/or health and wellbeing in primary and secondary schoolteachers.

**Table 4:** Summary of round-one e-Delphi consensus votes to ascertain the value of constructs and lifestyle covariates

Lifestyle Covariates/ Constructs	Consensus vote % (*n* = Total number of agree/mostly agree or include votes)	Disagree/ Exclude	Unsure	Blank
*Screening Constructs*				
Anthropometric	88 (*n* = 15)	2	6	0
Demographic	*Items within these constructs were reviewed separately by experts to refine the screening questions to be included.*			
Teacher characteristics				
Additional FN education				
School characteristics	79 (*n* = 11)	3	9	0
*FN Constructs*				
General health status and perceptions	100 (*n* = 22)	0	0	1
Perception of diet quality importance to health	95 (*n* = 20)	1	0	2
General food behaviours	95 (*n* = 20)	1	2	0
Weight perceptions, attitudes, and practices	86 (*n* = 18)	3	1	1
Eating and food behaviour at school	91 (*n* = 20)	2	1	0
School food preparation resources	95 (*n* = 19)	1	3	0
Meal sharing practices	95 (*n* = 19)	1	3	0
Home food preparation responsibilities	100 (*n* = 22)	0	1	0
Cooking attitudes and practices	100 (*n* = 21)	0	1	1
Cooking attitude and self-efficacy	95 (*n* = 19)	1	1	2
Food skills frequency/confidence	95(*n* = 19)	1	2	1
Perceptions of healthy eating support	95 (*n* = 18)	1	3	1
Eating social norms at school	94 (*n* = 16)	1	2	4
Dietary assessment (FAVVA Index)	80 (*n* = 16)	4	2	1
Resilience and resistance eating practices	94 (*n* = 15)	1	4	3
Teacher FN role and perception of confidence	95 (*n* = 19)	1	1	2
*Wellbeing Constructs*				
Personal wellbeing	85 (*n* = 17)	3	3	0
Individual quality of life	80 (*n* = 16)	4	3	0
School morale and teacher productivity	89 (*n* = 17)	2	4	0
Teacher work-related wellness	94 (*n* = 16)	1	4	2
Teacher work-related burnout	81 (*n* = 13)	3	3	4
Teacher work-related stress and coping	82 (*n* = 14)	3	3	3
*Lifestyle Covariates*				
Physical activity	91 (*n* = 20)	2	1	0
Sedentary behaviour	86 (*n* = 19)	3	1	0
Sleep	83 (*n* = 19)	4	0	0
Smoking and tobacco use	91 (*n* = 20)	2	0	1
Alcohol intake	100 (*n* = 22)	0	1	0
Food security	90 (*n* = 19)	2	2	0

Legend: FN = Food and Nutrition

II. Open-ended qualitative feedback from experts

Where consensus value was obtained but the proportion of votes indicating a ‘mostly agree’ response was higher (i.e., some amendments or item changes are required), qualitative feedback from experts was reviewed to make improvements including the removal of redundant, and/or repetitious constructs. Of the wellbeing constructs considered, experts identified that the Quality of life (QoL) [24], Personal Wellbeing [31], and Teacher Work-related Wellness [32] constructs would potentially increase participant response burden if all were included in the same questionnaire, without providing any new data.

III. Rank questions

Table 5 provides a detailed overview of the rank questions conducted with the results used in conjunction with expert qualitative feedback and consensus vote to guide constructs and LC for exclusion. Using rank questions and qualitative feedback the QoL [24], and Teacher Work-related Wellness [32] constructs were removed, with the four-item Personal Wellbeing [31] construct remaining. Further to this the LC for Smoking and Tabacco Use, Sedentary Behaviour and Food Security were removed.

**Table 5:** Summary of rank questions

Items	Top three constructs to Include	Top three constructs to Exclude
Food and nutrition constructs	• food skills frequency/confidence • eating behaviour at school • teacher role and perception of confidence	• weight perceptions attitudes and practices • resilience and resistance eating practices • meal sharing
Wellbeing constructs	• stress and coping • teacher work-related wellness • personal wellbeing	• morale • burnout • quality of life
Lifestyle covariates	• physical activity • alcohol intake • sleep quality/quantity	• smoking status/ tobacco use • sedentary behaviour • food security

IV. New constructs created for round-two

Expert feedback highlighted the need to refine the larger constructs proposed in round-one such as General Health Perceptions and Food Behaviours, and re-assemble the question items into smaller, more concise constructs with a single focus, including recommendations for the screening constructs of Demographic and Teacher Characteristics. Therefore, the living arrangements question from the Demographic construct was isolated and made into a standalone single-item construct, with the new construct of Food Avoidance created to focus on reasons of food avoidance including diagnosed allergy and intolerances. The questions from the General Food Behaviours group were re-assigned to create the Beverage Intake construct, with the meal frequency questions moved to the new Food Behaviours construct and the supplement questions removed altogether.

Data from participants who completed round-one but chose not to complete round-two, were incorporated into the consensus vote and analysis for round-one. However, participants who did not complete round-one, following a reminder email were not invited to participate in round-two.

Results from round-one were finalised and a second TFNQ version prepared.

### Research team meeting

Expert feedback was discussed and compared with results from the consensus votes and rank questions to determine further constructs and LC to be removed, adjusted and/or split. Additional file 2 provides a detailed summary of the constructs, and LC throughout the e-Delphi process with a summary of each, and the number of items included.

I. Constructs with conflicting expert feedback

While the Work-related Burnout [33] construct achieved consensus vote among experts as a work-related measure of wellbeing that would provide quality data, it was identified in the rank questions as a construct for potential removal alongside Teacher Work-related Wellness [32]. However, reflecting on emerging research showing associations between burnout and nutrition [20] it was decided it should remain, with the Teacher Work-related Wellness [32] construct removed. Additionally, in reviewing the measures proposed to assess teacher personal wellbeing, the Personal Wellbeing construct [31] and QoL [24] achieved similar scores in consensus vote and rank questions. However, in consideration of reducing participant response burden the four-item Personal Wellbeing [31] question group was selected over the 12-item QoL construct [24] moving into round-two. Meal Sharing Practices also achieved consensus as a construct to provide valuable information, but rank questions marked it for potential removal. It remained in the next stage of testing following researcher discussions due to potential social, psychological benefits and associations of meal sharing with wellbeing [34, 35].

II. New constructs for round-two

Of the FN constructs, General Food Behaviours was one that received extensive feedback from experts and required revision, with feedback identifying the potential to remove items on supplements, and rearrange questions on beverages, snacks, and takeaway into three separate constructs. While snacks and takeaway items remained within the new Food Behaviours construct in round-two, Beverage Intake was made into its own construct and included further category grouping within it following expert feedback to consider caffeinated style beverages, separately from sugar-sweetened beverages, and water.

### E-Delphi round-two

I. Consensus vote of question phrasing

Round-two focused on the non-validated constructs and LC specifically created for the TFNQ with 10 out of 17 achieving consensus to approve the item phrasing and scale style selected (Table 6). The remaining seven were just below the 75% cut point indicating minor adjustments to improve readability and clarity. Expert feedback was reviewed, and question items were added to the Food Rewards and Home Food Responsibilities constructs. With the phrasing amended in the Confidence of Food and Nutrition Roles, Beverage Intake, Teacher Characteristics, Food Avoidance, and Demographic constructs (See Additional file 2).

**Table 6:** Consensus vote summary on phrasing and scale suitability of constructs/ lifestyle covariates without previous validation

Lifestyle Covariates/ Constructs	Phrasing		Scale suitability	
	Consensus vote % (*n* = Total number of agree votes)	Disagree (*n* = Total number of blank or unsure votes)	Consensus vote % (*n* = Total number of agree votes)	Disagree (*n* = Total number of blank or unsure votes)
*Screening Constructs*				
Demographic	84 (*n* = 16)	3	80 (*n* = 12)	3 (*n* = 4)
Teacher characteristics	63 (*n* = 12)	7	67 (*n* = 8)	4 (*n* = 7)
Training and education	68 (*n* = 13)	6	100 (*n* = 13)	0 (*n* = 6)
Living arrangements	79 (*n* = 15)	4	60 (*n* = 9)	6 (*n* = 4)
*FN Constructs*				
Food avoidance	74 (*n* = 14)	5	86 (*n* = 12)	2 (*n* = 5)
Health perceptions	89 (*n* = 17)	2	87 (*n* = 13)	2 (*n* = 4)
Perception of diet quality for health	74 (*n* = 14)	5	79 (*n* = 11)	3 (*n* = 5)
School food resources	89 (*n* = 17)	2	86 (*n* = 12)	2 (*n* = 5)
Perceptions of healthy eating support	84 (*n* = 16)	3	100 (*n* = 15)	0 (*n* = 4)
Meal sharing practices	79 (*n* = 15)	4	100 (*n* = 15)	0 (*n* = 4)
Home food responsibilities	84 (*n* = 16)	3	80 (*n* = 12)	3 (*n* = 4)
Beverage intake	58 (*n* = 11)	8	93 (*n* = 14)	1 (*n* = 4)
Food behaviours	63 (*n* = 12)	7	93 (*n* = 14)	1 (*n* = 4)
Confidence of FN roles	89 (*n* = 17)	2	93 (*n* = 13)	1 (*n* = 5)
Food rewards	72 (*n* = 13)	5 (*n* = 1)	93 (*n* = 14)	1 (*n* = 4)
*Wellbeing Constructs*				
Workload	84 (*n* = 16)	3	92 (*n* = 11)	1 (*n* = 7)
*Lifestyle Covariates*				
Alcohol intake	89 (*n* = 16)	2 (*n* = 1)	87 (*n* = 13)	2 (*n* = 4)

II. Consensus vote of scale suitability

The suitability of scales was assessed with 15 out of 17 achieving consensus (Table 6). Two obtained a consensus below the 75% cut-point indicating improvements were needed, these being the Teaching Characteristics and Living Arrangements constructs which achieved 67% and 60% consensus score respectively. Expert suggestions were reviewed with amendments made to the scales used.

III. Questionnaire flow

Questionnaire flow received 86% consensus with only minor feedback resulting in two constructs being split, these being Meal Sharing Practices at school/ home and Perceptions of Healthy Eating Support at school/home. This change allowed the school and home focused questions to be clustered to improve participant flow through questions.

## Discussion

FN factors that impact an individual’s daily eating practices are complex, with 441 determinants, (i.e., factors that influence food, nutrition and eating practices of individuals) identified across 51 groups within the original DONE framework [36]. The complexity of measuring FN was considered when developing the TFNQ, along with the responsibilities of teachers as FN Influencers to ensure constructs and LC selected would provide valuable data on both personal and professional FN factors in relation to overall teacher health and wellbeing. Tools measuring FN constructs have been identified globally, including those used in teacher populations, although few tools have included a variety of FN constructs, with inconsistency across descriptions and psychometric testing to assess validity [6]. A questionnaire that includes a range of FN constructs to address this complexity in the context of teacher FN responsibilities is essential to enable the comprehensive evaluation of teacher FN-related health and wellbeing.

To the best of our knowledge the TFNQ is the first questionnaire specifically designed to measure FN as a component of health and wellbeing among primary and secondary schoolteachers. All the food, nutrition, health, and wellbeing constructs and LC included within the initial TFNQ achieved a consensus score of 80% or above, identifying that all would potentially provide valuable data to measure aspects of teacher FN-related health and wellbeing. Each construct and LC were selected with consideration of the association with either diet quality, and/or aspects of teacher wellbeing and of the professional FN roles teachers undertake as FN Influencers. The expert e-Delphi assisted to establish content validity of the TFNQ and in refining unnecessary or repetitious constructs to reduce user response burden and ensure valuable data to measure teacher FN-related health and wellbeing.

### Diet quality within the TFNQ

Diet quality is recognised globally as a correlate of key health outcomes [18], with recent studies exploring its association with mental health and wellbeing outcomes [14]. Dietary intake is often the most frequently assessed construct in nutrition interventions, including those with teacher participants [6, 17], with self-reported methods using food frequency questionnaires or brief dietary screeners. The FAAVA diet quality index [22] included within the TFNQ is a dietary screener with established comparative validity that provides a measure of diet quality in the frequency and variety of fruit and vegetable consumption. Inclusion of a diet quality measure within the TFNQ enables the exploration of potential associations with other constructs such as Food Skills Confidence [25], a previously validated construct, used within an Australian study that demonstrated a correlation with diet quality [37]. Higher teacher diet quality scores have been shown previously to be associated with healthier nutrition-related classroom practices [9], where teacher diet quality was a marginally significant predictor of classroom food practices, including use of food rewards [8].

Teacher participation in food preparation responsibilities is another food construct included in the TFNQ to further explore associations between individual engagement with home food preparation responsibilities and diet quality [38]. Additionally, there is interest in exploring connections between home food preparation and cooking practices, including aspects of wellbeing [16, 39] or as a potential self-coping strategy to manage anxiety [40]. Teacher time spent in food preparation has also been used in teacher fidelity evaluations of student nutrition interventions where a relationship was observed between time teachers spent in self-food preparation and their reported self-efficacy or comfort of FN program delivery [41].

### Wellbeing constructs within the TFNQ

A variety of wellbeing constructs are included in the TFNQ to measure different aspects of teacher personal and work-related wellbeing including, personal subjective wellbeing [31], stress, coping [42] and burnout [33]. The 2022 Australian Principals Survey identified teacher shortages within their top three stress concerns, with stress experienced in relation to dealing with the mental health and wellbeing of students and staff ranking 4th and 5th respectively [43]. Further, the top four staff-related concerns Principals reported were teacher burnout, stress, anxiety, and depression issues [43]. Therefore, to ensure a comprehensive assessment of wellbeing the TFNQ includes these four key wellbeing measures of Work-related Burnout [33], Personal Wellbeing [31] and Work-related Stress and Coping [42]. Despite research indicating potential correlations between level of burnout experienced and diet quality [20], wellbeing research in teachers is limited in its exploration of this [5, 7]. Hence, the TFNQ is designed to be an inclusive measure of wellbeing and FN to allow these relationships to be further explored.

Subjective wellbeing (measured within the Personal Wellbeing [31] construct in the TFNQ) is observed across teacher-related studies as a predictor of teacher stress, with noted associations with social support (i.e., school leadership support and school climate), as well as teacher effectiveness and self-efficacy outcomes [44]. The Australian Teacher Workforce Database has highlighted in 2021, that of the 38% of teachers indicating an intention to leave the profession before retirement, 87% of them acknowledged stress and coping as the main reasons, with 68% identifying the impact of stress on their overall mental health and wellbeing [45]. The single-item questions measuring stress and coping within the TFNQ have been validated in a teacher population with significant correlation between coping, burnout and emotional exhaustion recognised, where caution is noted in assessing coping independently of stress [42].

Teacher wellbeing should not be viewed in light on individual factors alone, due the influence of colleagues, school leaderships and the school environment noted within teacher qualitative studies as potential barriers or facilitators to achieving healthy wellbeing practices [21]. Therefore, TFNQ also includes an Eating Social Norms at School construct [23] and a social support construct that measures teacher perceived social support from home and school life to investigate their influence on teacher FN wellbeing with social support described as a potential correlate of teacher anxiety levels and depression in previous research [46].

Finally, Meal Sharing Practices at home and school was included as a construct within TFNQ given potential associations with wellbeing and diet quality. Meal sharing practices are often discussed in research with reference to its impact on diet quality and psychological outcomes, with most studies looking at the connection with at-home meals and the impact on child and adolescent health [47]. Studies within teacher populations have acknowledged the benefit of shared meals to promote teacher support, facilitate channels of open communication between peers [48] and benefit wellbeing [35]. Additionally, a call from recent reviews identifies the general need for stronger evaluation tools and study designs to better investigate the relationship of meal sharing practices in relation to diet quality and wellbeing outcomes [34].

### Confidence of food and nutrition roles and food rewards constructs

Teacher wellbeing attitudes, including food preparation habits and commitments have been shown to influence how a teacher may value the importance of nutrition education and level of program engagement [3, 41]. These attitudes and beliefs can influence the curriculum content teachers choose to reinforce [49] or play a role in shaping teacher classroom food practices, including the use of food rewards [8]. While teacher FN beliefs and attitudes are not directly measured within the TFNQ, teacher Food Skills Confidence [25], Food Preparation Practices, and dietary intake (using the FAVVA index) [22] are included to investigate these relationships further. Consideration is also given to how these constructs may influence teacher confidence or self-efficacy in undertaking their professional FN responsibilities as role models, educators, and policy implementors which are to be measured with the Confidence of FN Roles and Food Rewards constructs.

### Nutrition knowledge

Nutrition knowledge is used across many teacher-related studies as a correlate with nutrition education self-efficacy and classroom practices [3]. However, due to variation across school systems in Australia and globally regarding what is taught in relation to FN including a nutrition knowledge component to measure associations with teacher professional FN practices was not considered at this stage. Previously validated measures to assess general nutrition knowledge exist for the Australian population [50] but inclusion would have increased questionnaire length and participant completion burden without adding to overall data. Knowing what to eat does not translate into positive behaviours due to conflicting barriers [51], therefore, as nutrition knowledge is acknowledged as a weak correlate of diet quality and health-related outcomes in adults [19], it was not selected for inclusion within the TFNQ.

### Lifestyle covariates in the TFNQ

When measuring wellbeing it is important to review key LC, with sleep [52], diet quality [22] and physical activity [53, 54] being the main three to be included within the final TFNQ. A recent review highlighted that of the three, sleep, and especially sleep quality, was identified to have a strong relationship with wellbeing outcomes [55]. Additionally, it was identified that within the context of diet, particular associations are noticed between fruit and vegetable intake and wellbeing outcomes [55].

### Potential impact of this research

Beneficiaries of teacher wellbeing programs are two-fold, with the potential to improve both health and wellbeing outcomes of teachers as well as students. Supporting teachers to improve their FN practices as part of an integrative wellbeing approach could be the key to achieving a positive shift in the current teacher wellbeing situation, with follow-on effects in the self-efficacy of teachers in fulfilling their professional FN responsibilities as role models, gate keepers and health promoters.

Teachers are Influencers of FN practices within the school environment and identified in student focused research acknowledging that students are often observant of teacher FN practices [56]. Teachers charged with implementing health programs and delivering nutrition education have better program buy in and teaching self-efficacy when teacher professional development is included within the intervention or where there is provision of nutrition education available to support teachers [3]. The recommendations of global organisations like the United Nations Educational Scientific and Cultural Organization and World Health Organization, echo these sentiments by highlighting the need for both pre-service teacher training and ongoing professional development for teachers and other school staff to better enable the health promoting capacity of schools [57]. Therefore, having a validated screening tool readily available that enables effective evaluation of teacher FN could improve the quality of data collected and allow detailed evaluation of the status of teacher FN and its relationship with overall teacher health and wellbeing.

### Limitations

The online format used to conduct the e-Delphi study allowed the participation of a wide range of international and local experts across education, dietetics and nutrition, FN behaviour, and psychology, to determine the ability of TFNQ content to measures the influence of FN as a component of teacher health and wellbeing. However, the privacy software of expert institutional emails caused difficulties with distribution and receipt of the round-two survey and may have contributed to a reduced number of expert responses observed.

Additionally, the diversity of experts, while an asset, was the reason for including an unsure option within the consensus vote for questions beyond the scope of a participant’s expertise, therefore the number of votes provided on some aspects throughout the e-Delphi may have been impacted by the balance of unsure votes. It should be acknowledged that the findings of this study report the development of the TFNQ and identify the process of establishing content validation conducted through the expert e-Delphi. Future studies should consider psychometric testing to assess the reliability of the TFNQ and additional construct validity to evaluate its ability to measure the FN-related health and wellbeing of primary and secondary schoolteachers.

## Conclusions

A validated questionnaire, applicable for use in quality data collection could facilitate research while also providing a tool to inform teacher education efforts at both undergraduate training and within continuing professional development of practicing teachers. To date, there are no teaching standards or policy provision within Australia that identify FN knowledge and skills all primary and secondary teachers need to effectively support their own wellbeing, and/or in being positive Influencers of FN, as role models, health promoters, advocates, and gatekeepers within schools. The TFNQ will be used to obtain data that aims to inform education interventions and guide policies that support teachers to maintain positive FN practices while being effective FN Influencers to the many students in their care.

## Electronic supplementary material

Below is the link to the electronic supplementary material.

Additional file 1: Glossary of key words (pdf format)

Additional file 2: A summary of the changes to constructs and lifestyle covariates throughout the e-Delphi process (pdf format)

## Data Availability

Data is provided within the manuscript or supplementary files. The TFNQ can be made available upon request to corresponding author.

## Abbreviations

FN: Food and Nutrition
TFNQ: Teacher Food and Nutrition-related health and wellbeing Questionnaire
DONE Framework: The Determinants Of Nutrition and Eating Framework
LC: Lifestyle Covariate
FAVVA: Fruit and Vegetable VAriety Index
QoL: Quality of Life
DQ: Diet Quality
R1: Round One
R2: Round Two
