# Supplementary material for: Content validation of the teacher food and nutrition-related health and wellbeing questionnaire, a Delphi study
Source: BMC Public Health. 2025 Apr 21;25:1468. doi: 10.1186/s12889-025-22555-0 (PMC12010570; doi:10.1186/s12889-025-22555-0)
Supplement: Supplementary file 1 — Additional file 1: Glossary of key words (pdf format) [file 12889_2025_22555_MOESM1_ESM.pdf]

Additional file 1: Glossary of key words

GLOSSARY

Covariates: independent variables (i.e., physical activity, sleep, and alcohol intake) included for review of their potential contributions to the outcomes of interest.

Correlate: variable (construct) with an identified mutual relationship to the outcomes of interest

Construct: used within this study to denote a scale or group of questions that measure an aspect of food, nutrition, health, or wellbeing

Determinant: defined within the DONE Framework as the many factors, including those outlined within the DONE framework, that influence the food, nutrition and eating practices of individuals [(1, 2)].

Food and/or nutrition <u>factor:</u> a known determinant or aspect of influence that may impact an individual's ability to make healthy FN choices, used in this study as a general term to capture any attitude, behaviour, motivation, skill, level of knowledge etc that can influence an individual's FN choices and practices.

Nutrition education (teaching) self-efficacy: Teacher confidence to deliver and teacher nutrition education or implement aspects of nutrition interventions.

Personal Teacher FN: factors related to an individual teacher, even though these may have downstream effects on student FN factors i.e., diet quality, food skills, food attitudes and perceptions.

Personal FN Constructs: a group of questions, or scale that measure an aspect of teacher personal health and wellbeing such as dietary intake, food and cooking skills, food attitudes and behaviours.

Additional file 1: Glossary of key words

Professional FN: factors specifically related to a teachers' professional role, even though these may potentially also influence personal FN i.e., classroom FN practices, role modelling food behaviours, health promotion and FN educator.

Professional FN Constructs: a group of questions, or scale that measure a teacher's professional FN attitudes, knowledge, or practices such as classroom FN practices, nutrition teaching self-efficacy or capacity to role model healthy FN practices.

References

1. Stok FM, Hoffmann S, Volkert D, Boeing H, Ensenauer R, Stelmach-Mardas M, et al. The DONE framework: creation, evaluation, and updating of an interdisciplinary, dynamic framework 2.0 of determinants of nutrition and eating. PLoS One. 2017;12(2):e0171077.
2. DEDIPAC (Determinants of Diet and Physical Activity) Knowledge Hub. The DONE (Determinants of Nutrition and Eating) interactive framework. A DEDIPAC knowledge hub output [cited January 2024]. Available from: https://www.uni-konstanz.de/DONE/
