# Supplementary material for: Content validation of the teacher food and nutrition-related health and wellbeing questionnaire, a Delphi study
Source: BMC Public Health. 2025 Apr 21;25:1468. doi: 10.1186/s12889-025-22555-0 (PMC12010570; doi:10.1186/s12889-025-22555-0)
Supplement: Supplementary file 2 — Additional file 2: A summary of the changes to constructs and lifestyle covariates throughout the e-Delphi process (pdf format) [file 12889_2025_22555_MOESM2_ESM.pdf]

Additional file 2- A summary of the changes to constructs and lifestyle covariates throughout the e-Delphi process

**Initial Questionnaire <u>Round One</u>**		**<u>Round Two</u> Questionnaire**		**<u>Preliminary</u> Questionnaire**<br>*Changes made from final expert comments*	
Constructs/LC	Description	Constructs/LC	Description	Constructs/LC	Description
**Screening Constructs**					
Anthropometric (n=2)	Self-reported<br>• Weight<br>• Height				
Demographic (n=6)	• Year of birth<br>• Gender<br>• Primary ethnicity<br>• Aboriginal and Torres Strait Islander origin<br>• Current living arrangements	Demographic (n=5)	• Year of birth<br>• Gender<br>• Primary ethnicity<br>• Aboriginal and Torres Strait Islander origin	Demographic (n=5)	• Year of birth<br>• Gender<br>• Primary **ancestry**<br>• Aboriginal and Torres Strait Islander origin
		Living arrangements (n=1)<br>*From: Demographic question group R1*	• Current living arrangements	Living arrangements (n=1)	• **Typical** living arrangements
Teacher characteristics (n=12)	• Status and contract type<br>• Additional work hours<br>• Training<br>• Experience<br>• Primary and additional roles<br>• Level and subject area taught	Teacher Characteristics (n=5)	• Status and contract type<br>• Level and subject area taught<br>• Primary role	Teacher Characteristics (n=5)	• Status and contract type<br>• Level and subject area taught<br>• Primary role
Additional food and nutrition education (n=3)	• Food and nutrition professional development<br>• Other food and nutrition training	Qualifications and education (n=3)	• Training<br>• Nutrition subject in your teacher training	**Education** (n=4)	• Training<br>• Nutrition subject in your teacher training

Additional file 2- A summary of the changes to constructs and lifestyle covariates throughout the e-Delphi process

**Initial Questionnaire Round One**		**Round Two Questionnaire**		**Preliminary Questionnaire**<br>*Changes made from final expert comments*	
Constructs/LC	Description	Constructs/LC	Description	Constructs/LC	Description
			• Professional development or other study		• Professional development or other study
School characteristics (n=1)	• Name<br>• State	School characteristics (n=1)	• Name<br>• State	School characteristics (n=1)	• Name<br>• State
**Food and Nutrition Constructs**					
General health status and perceptions (n=6)	• General health<br>• Chronic disease and mental health status<br>• Allergies/intolerance<br>• Food avoidance	*Items removed (n=3)*<br>*Others became two new question groups:*<br>• Health perceptions<br>• Food avoidance			
		Health perceptions (n=2)<br>*From: General health status and perceptions question groups R1*	• Physical health<br>• Mental health	Health perceptions (n=2)	• Physical health<br>• Mental health
Perception of diet quality (DQ) importance to health (n=4)	• Perception DQ<br>• Importance of DQ to:<br>○ Physical health<br>○ Mental health<br>• Overall wellbeing	Perception of diet quality for health (n=3)	• Perception of DQ<br>• Importance of DQ to:<br>○ Physical health<br>○ Mental health	Perception of diet quality for health (n=3)	• Perception of DQ<br>• **Impact** of DQ on:<br>○ Physical health<br>○ Mental health
				Dietary pattern (n=1)	Usual eating pattern
		Food avoidance (n=4)	• Allergy/intolerance<br>• Foods avoided<br>• Reasons for food avoidance	Food avoidance (n=3)	• Allergy/intolerance<br>• Foods avoided<br>• Reasons for food avoidance

Additional file 2- A summary of the changes to constructs and lifestyle covariates throughout the e-Delphi process

**Initial Questionnaire Round One**		**Round Two Questionnaire**		**Preliminary Questionnaire**<br>*Changes made from final expert comments*	
Constructs/LC	Description	Constructs/LC	Description	Constructs/LC	Description
		From: General health status and perceptions question groups R1			
General food behaviours (n=9)	• Supplements<br>• Meal frequency<br>• Beverage intake (water, soft drink)	Beverage intake (n=4)<br><br>*Items removed or transferred to school food behaviours, with others added (caffeine)*	Intake of caffeine, water, and high energy drinks	Beverage intake (n=4)	• Intake of caffeine, water, and high energy drinks<br>• Type of beverage
				Food behaviours<br><br>*Meal frequency removed from school food behaviours R2 and expanded here.*	• Main meal frequency of intake (n=3)<br>• Take away frequency<br>• Common take away foods
Weight perceptions, attitudes, and practices (n=4)	• Weight change attempts<br>• Current practice<br>• Weight goals<br>• Weight perceptions				
Eating and food behaviour at school (n=6)	• Frequency of lunch intake<br>• Work/school barriers<br>• Location	Food behaviours (n=4)<br><br>*Some items removed and others added from general*	Frequency of breakfast, lunch, takeaways, and snack intake	Eating and food behaviour at school (n=2)	• Snack frequency<br>• Common snack foods

Additional file 2- A summary of the changes to constructs and lifestyle covariates throughout the e-Delphi process

**Initial Questionnaire Round One**		**Round Two Questionnaire**		**Preliminary Questionnaire** *Changes made from final expert comments*	
Constructs/LC	Description	Constructs/LC	Description	Constructs/LC	Description
		food behaviours group R1			
School food preparation resources (n=5)		School food preparation resources (n=3)		School food **resources** (n=3)	
Meal sharing practices (n=3)	• Home<br>• Work/school	Meal sharing practices (n=2)	• Home<br>Work/school	Meal sharing practices (n=1)	Home
				Meal sharing practices (n=1)	Work/school
Home food preparation responsibilities (n=3)	• Food shopping<br>• Meal planning<br>• Meal preparation	Home food preparation responsibilities (n=3)	• Food shopping<br>• Meal planning<br>•Meal preparation	Home food preparation responsibilities (n=5)	• Food shopping<br>• Meal planning<br>• Meal preparation<br>o Breakfast<br>o Lunch<br>o Dinner
Cooking attitudes and practices (n=3)	• Cooking attitude<br>• Frequency of home food preparation<br>• Time spent preparing a meal				
Cooking attitude and self-efficacy (n=10)	• Self-efficacy and attitudes subscale of the CAFPAS-Cooking and food provisioning action scale, to assess individual food agency				
Food skills frequency/ confidence (n=19)	• Menu planning and preparing<br>• Shopping	Food skills frequency/ confidence (n=19)	• Menu planning and preparing<br>• Shopping	Food skills frequency/ confidence (n=19, *split into 5* categories)	• Menu planning and preparing<br>• Shopping

Additional file 2- A summary of the changes to constructs and lifestyle covariates throughout the e-Delphi process

**Initial Questionnaire Round One**		**Round Two Questionnaire**		**Preliminary Questionnaire**<br>*Changes made from final expert comments*	
Constructs/LC	Description	Constructs/LC	Description	Constructs/LC	Description
	• Budgeting<br>• Resourcefulness<br>• Label reading/ consumer awareness		• Budgeting<br>• Resourcefulness<br>• Label reading/ consumer awareness		• Budgeting<br>• Resourcefulness<br>• Label reading/ consumer awareness
Perceptions of healthy eating support (n=2)	• School culture<br>• Home/family	Perceptions of healthy eating support (n=4)	At school<br>• School culture<br>• Colleagues<br>At home<br>• Home/family<br>• Friends	Perceptions of healthy eating support (n=2)<br><br>*Separated within the questionnaire to allow grouping of and home questions for better logic flow.*	At school<br>• School culture<br>• Colleagues
					At home<br>• Home/family<br>• Friends
Eating social norms at school (n=7)		Eating social norms at school (n=7)		Eating social norms at school (n=7)	
Dietary assessment ~ fruit and vegetable screener (n=35)	• Diet quality (FAVVA index)	Dietary assessment ~ fruit and vegetable screener (n=35)	Diet quality (FAVVA index)	Dietary assessment ~ fruit and vegetable screener (n=35)	Diet quality (FAVVA index)
Resilience and resistance eating practices (n=6)					
Teacher food and nutrition role and perception of confidence (n=6)	• Role model<br>• Educator<br>• Health promoter<br>• Conversations<br>• Gate keeper (provision of food rewards)	Confidence of food and nutrition roles (n=4)	• Role model<br>• Educator<br>• Health promoter<br>• Conversations	Confidence of food and nutrition roles (n=4)<br><br>Food rewards made into a new construct	• Role model<br>• Educator<br>• Health promoter<br>• Conversations

Additional file 2- A summary of the changes to constructs and lifestyle covariates throughout the e-Delphi process

**Initial Questionnaire Round One**		**Round Two Questionnaire**		**Preliminary Questionnaire**<br>*Changes made from final expert comments*	
Constructs/LC	Description	Constructs/LC	Description	Constructs/LC	Description
		Food rewards (n=1)	• Frequency	Food rewards (n=2)	• Food reward<br>• Common food rewards
**Wellbeing constructs**					
Personal wellbeing (n=4)	Subjective wellbeing<br>• Satisfied (Evaluative)<br>• Worthwhile (Eudaemonic)<br>• Happy (Hedonic/positive)<br>• Anxious (Hedonic/negative)	Personal wellbeing (n=4)	Subjective wellbeing<br>• Satisfied<br>• Worthwhile<br>• Happy<br>• Anxious	Personal wellbeing (n=4)	Subjective wellbeing<br>• Satisfied<br>• Worthwhile<br>• Happy<br>• Anxious
Individual quality of life (n=12)					
School morale and teacher productivity (n=2)	• Morale<br>• Absenteeism	Productivity (n=1)	Absenteeism	Productivity (n=1)	**Sick leave**
Teacher work-related wellness (n=4)					
Teacher work-related burnout (n=4)	Emotional exhaustion<br>• Drained<br>• Depleted<br>• Fatigued<br>• BurntOut	Teacher work-related burnout (n=4)	Emotional exhaustion<br>• Drained<br>• Depleted<br>• Fatigued<br>• BurntOut	Teacher work-related burnout (n=4)	Emotional exhaustion<br>• Drained<br>• Depleted<br>• Fatigued<br>• BurntOut
Teacher work-related stress and coping (n=2)	• Stress<br>• Coping	Teacher work-related stress and coping (n=2)	• Stress<br>• Coping	Teacher work-related stress and coping (n=2)	• Stress<br>• Coping
		Workload (n=1)	Additional hours of work/per week	Workload (n=1)	Additional hours of work/per week

Additional file 2- A summary of the changes to constructs and lifestyle covariates throughout the e-Delphi process

**Initial Questionnaire Round One**		**Round Two Questionnaire**		**Preliminary Questionnaire**<br>*Changes made from final expert comments*	
Constructs/LC	Description	Constructs/LC	Description	Constructs/LC	Description
**Lifestyle covariates**					
Physical activity (PA) (n=3)	• Time in PA<br>• Days of PA<br>• Strengthening	Physical activity (PA) (n=2)	• Time in PA<br>• Days of PA	Physical activity (PA) (n=2)	• Time in PA<br>• Days of PA
Sedentary behaviour (n=3)					
Sleep (n=2)	• Quality<br>• Quantity	Sleep (n=2)	• Quality<br>• Quantity	Sleep (n=2)	• Quality<br>• Quantity
Smoking and tobacco use (n=3)	• Frequency<br>• Amount				
Alcohol intake (n=2)	• Frequency<br>• Amount	Alcohol intake (n=2)	• Frequency<br>• Amount<br>*Image added to question to demonstrate standard drink.*	Alcohol intake (n=2)	• Frequency<br>• Amount
Food security (n=2)	• Security<br>• Resources				

Legend: R1= Round One, R2=Round Two, FAVVA index= Fruit And Vegetable VAriety index, LC= Lifestyle covariate, FN=Food and Nutrition, PA= Physical Activity, DQ= Diet Quality, n=no’ items in each measure
